# Plasma ACTH concentration and pituitary gland histo-pathology in rats infected with *Trypanosoma brucei brucei*

**DOI:** 10.4314/ahs.v17i4.10

**Published:** 2017-12

**Authors:** Charles Irungu Maina

**Affiliations:** Egerton University, Department of Biological Sciences, P.O. Box 536, 20115-Egerton, Kenya. cimainah@gmail.com Tel; +254728425209

**Keywords:** Trypanosomiasis, paraventricular nucleus, pituitary gland, ACTH

## Abstract

**Background:**

Human African trypanosomiasis is one of the neglected and re-emerging infectious diseases in Africa with over 60 million people being at risk of contracting the disease.

**Objective:**

To investigate the effects of *Trypanosoma brucei brucei* infection on secretion of adrenocorticotropic hormone (ACTH) and histology of the pituitary gland and paraventricular nucleus in rats.

**Methods:**

Rats were randomly divided into two groups, control and experimental. Experimental rats were injected intraperitonially with 0.2ml of blood containing 1.0 × 10^4^ live *T.b.brucei* parasites. Tail blood samples were collected weekly for the determination of plasma concentration of ACTH. The pituitary gland and coronal section of brain were processed histologically and observed microscopically.

**Results:**

There was a significant difference (p = 0.0190) in plasma ACTH concentration between the control and experimental rats. Histological alterations were observed in both the pituitary and paraventricular nucleus of experimental rats.

**Conclusion:**

*T.b.brucei* infection causes histological changes in both the paraventricular nucleus and pituitary gland in rats. These histological changes could account for the decrease in corticotropin releasing hormone (CRH) and ACTH production in the infected rats.

## Introduction

Human African trypanosomiasis (HAT), better known as sleeping sickness, is one of the neglected and re-emerging infectious diseases in Africa. Over 60 million people are at risk of contracting the disease, and 300,000 to 500,000 cases are reported annually, with at least 60,000 deaths^1^. The disease is caused by protozoan parasites of the genus *Trypanosoma* and is transmitted by the bite of an infected *tsetse fly* of the genus *Glossina*.

There are two forms of HAT; a chronic form caused by *Trypanosoma brucei gambiense*, which occurs in West and Central Africa, and the acute form, caused by *T.b. rhodesiense*, which occurs in Eastern and Southern Africa^2^. There are also two distinct stages of disease progression. During the early, or haemolymphatic, stage, the parasites invade and spread within the blood, lymphatic system and systemic organs. After about 3 weeks, the disease progresses to the late, or neurological, stage, where the parasites cross the blood-brain barrier to enter the central nervous system^3^. Once the central nervous system is invaded the patient may suffer from a constellation of neurological symptoms and signs including motor, psychiatric and sensory disorders and disruption of normal sleep/wake cycle. The symptoms of the second stage give the disease its other name, sleeping sickness. If untreated or inadequately treated, all patients with HAT eventually die.

The causative agents of trypanosomiasis, the *Trypanosoma* parasites , show early invasion in brain areas that lack a blood-brain barrier, such as the pineal gland and median eminence^4^. From here, the trypanosomes invade other brain regions including the hypothalamus and the pituitary gland where they cause inflammatory responses that may lead to disruptions in endogenous circadian rhythms including hormone secretion. The purpose of this study was to investigate the effects of *T.b.brucei* on the secretion of adrenocorticotropic hormone (ACTH) and histology of the pituitary gland and paraventricular nucleus in rats.

## Materials and methods

### Experimental Setup

Twenty four male albino rats, aged 3–3½ months and weighing 200–220g, were used in this study. The rats were randomly divided into two groups, control and experimental, of twelve rats each. The rats were housed at room temperature in the mini-laboratory animal house in the Department of Biological Sciences, University of Eldoret, Kenya, where the study was carried out. They were housed three per cage and were exposed to 12/12 hours of light/dark cycle throughout the study period. The rats had access to food (mice pencil, Unifeed Millers Ltd, Kisumu, Kenya) and clean water *ad libitum*.

Two weeks prior to data collection, the rats were observed and accustomed to routine handling. They were also screened for ectoparasites and each rat was injected subcutaneously with 0.01ml of Ivermectin (Ivermin®, Sinochem Ningbo Ltd., China), a broad spectrum parasiticide that effectively controls both ectoparasites and endoparasites^5^.

### Infection of experimental rats

An isolate of the parasite *T.b. brucei* (ILT at 1.4) was obtained from the International Livestock Research Institute (ILRI), Nairobi, Kenya. The parasite was originally obtained from the blood of a naturally infected cow in Uhombo, Kenya. The isolate was injected intraperitoneally into a donor rat for the purpose of expanding the stabilate for subsequent inoculation into the experimental group rats. The donor rat was put in a cage and transported to the animal house at the University of Eldoret.

The donor rat was monitored for the presence of parasites daily by direct microscope observation of trypanosomes in wet smears of blood samples obtained from tail bleeds. When parasitaemia was established five days post-infection, the donor rat was anaesthetised with ether and 2ml of blood obtained from it through cardiac puncture. One millilitre (1ml) of this blood was diluted with 2ml of phosphate buffered saline solution (pH 7.4). Then, 0.2ml of this blood, containing about 1.0 × 10^4^ live *T.b. brucei* parasites was injected intra-peritoneally to each of the twelve rats in the experimental group. The number of parasites was determined using the Neubauer haemocytometer method^6^. Rats in the control group were, concurrently, injected intra-peritoneally with 0.2ml normal saline.

### Determination of Plasma Concentration of ACTH

Tail blood samples were collected weekly (at 0800 hours) for the determination of plasma concentration of ACTH. Using the tail snip method, about 1ml of blood from each of the 24 rats was collected in a vacutainer coated with ethylene diamine tetra acetate (EDTA). The blood sample was immediately placed in a centrifuge (Centurion Scientific Ltd., UK) and centrifuged for 5 minutes at 12,000 revolutions per minute. The plasma was quickly decanted into a test tube and stored immediately in a chest freezer (Haier Electrical Appliances Inc., Philippines). The concentration of ACTH in the plasma was determined shortly after using a fully automated immunoanalyzer machine of excellent sensitivity and specificity of results (VIDAS®, bioMerieux SA, France).

### Organ Harvesting and Histological Studies

All the twelve infected experimental rats were allowed to go through the full course of infection and sacrificed when they were *in extremis*. For every experimental rat sacrificed, a control rat was sacrificed too. Each rat was anaesthetized with ether and then decapitated. A firm cut along the midline of the skull (through both parietal and frontal bones) was made using a sharp knife. Both parietal and frontal bones were tilted thus exposing the brain. The brain was then gently lifted out of the skull and immediately put in 10% buffered neutral formalin where it was fixed for at least 48 hours.

One week later, three brains were randomly selected from each group for further processing. Each of the selected brains was removed from the formalin solution and the pituitary gland extracted using a sharp surgical blade. A coronal section of the brain was also made. The pituitary gland and the coronal section were processed histologically using an automated tissue processor (Global Medical Instrumentation Inc., USA). Paraffin blocks were sectioned with a manual rotary microtome (Leica Biosystems, Germany) at 5µm thickness. The thin sections of the coronal section and the pituitary gland were mounted on glass slides and stained using the standard staining technique of haematoxylin and eosin^7^. The stained slides were observed under a light microscope (Euromex, Holland) and photomicrographs taken using a camera (Canon EOS, Canon Inc., Japan) attached to the microscope.

## Results

### Parasite detection and physical observation of the rats

Parasites were detected in the tail blood of experimental rats five to eight days post-infection. The experimental rats showed no signs of disease for the first fifteen days post-infection. Thereafter, they showed apparent fatigue, decreased activity, lack of appetite, discharge from the eyes and nose, and paralysis of limbs and tail. On the other hand, the control rats showed no signs of infection throughout the study period. They were also of normal behaviour, appetite, and general activity.

### Plasma ACTH concentration

**Figure 1:**
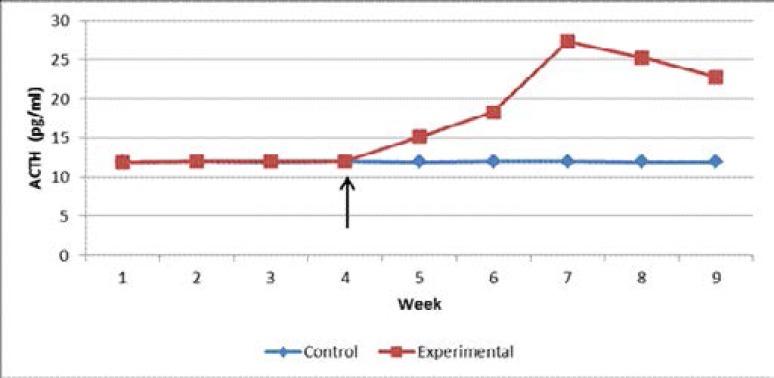
Pre- and post-infection plasma ACTH concentration in control and experimental rats. Arrow indicates time when experimental rats were inoculated with the *T.b.brucei* parasite.

The concentration of ACTH in the plasma of control rats ranged between 11.95 ± 0.10 pg/ml and 12.00 ± 0.10 pg/ml during the study period. Similarly, the concentration of ACTH in the plasma of experimental rats ranged between 11.90 ± 0.10 and 12.00 ± 0.15 pg/ml during the pre-infection period. However, the concentration rose steadily (up to 27.35 ± 0.10 pg/ml) for the first three weeks after infection and then decreased (up to 22.8 ± 0.35 pg/ml) at the end of the study period (Figure 1). The difference in plasma ACTH concentration between the control and experimental rats was significant (p = 0.0190; t-test).

### Paraventricular nucleus histology

**Figure 2:**
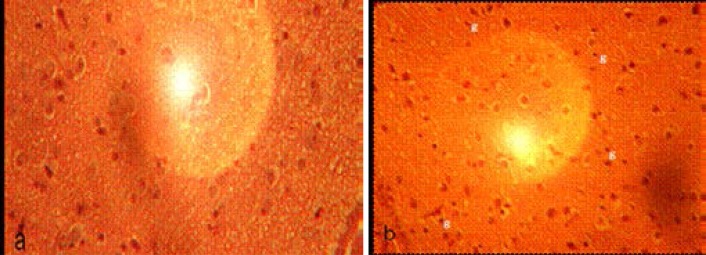
Photomicrograph of coronal section through the paraventricular nucleus of (a) control, and (b) experimental rats. (g, glial cells). Note the increased infiltration of glial cells in the experimental rat. Mg x400.

The paraventricular nucleus (PVN) of control rats showed normal neurons and fewer glial cells (Figure 2). The PVN of experimental rats showed neurons with smaller round nuclei and increased cellularity of glial cells. Tissue degeneration was not observed in this region of the brain.

### Anterior pituitary gland histology

**Figure 3:**
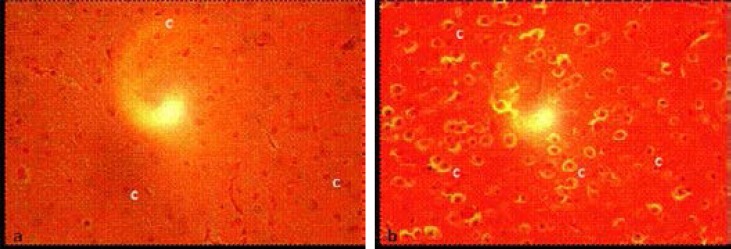
Photomicrograph of transverse section through the anterior pituitary gland of (a)control, and (b) experimental rats. (c, corticotroph). Mg x400.

The anterior pituitary of control rats showed normal corticotrophs with clearly visible round nucleus (Figure 3a). On the other hand, the anterior pituitary of experimental rats showed corticotrophs that had hypertrophied and then shrunk. Tissue degeneration was, however, not observed (Figure 3b).

## Discussion

The PVN is one of the neuronal nuclei in the hypothalamus. It is highly vascularised and is protected by the blood-brain barrier, although its neuroendocrine neurons extend to sites beyond the blood-brain barrier, notably, in the median eminence. The PVN contains multiple subpopulations of neurons that are activated by a variety of stressful and/or physiological changes.

In this study, there was a marked infiltration of glial cells in the PVN of experimental rats which could have been as a result of an immune response to trypanosome infection. This finding concurs with that of Chianella et al.^8^ who reported a remarkable activation of glial cells in the hypothalamus of trypanosome-infected rats. Human pathological studies have also reported an increase in glial cells in the brains of trypanosome-infected individuals^9^,^10^. Glial cells play a key role in the events triggered by infection; they not only serve as scavenger elements but also can assume phagocytic and cytotoxic properties in the very early stages of the immune response, can present antigens to T lymphocytes, and can even secrete cytokines^11^,^12^. In concurrence with an earlier study^8^, no degenerative changes were observed in the PVN of the trypanosome-infected rats in the present study.

Neurosecretory cells in the PVN synthesize and secrete corticotropin-releasing hormone (CRH) which is secreted into the hypothalamo-hypophyseal portal system and carried to the anterior pituitary gland where it stimulates the release of ACTH. ACTH reaches the adrenal cortex through the systemic circulation where it interacts with receptors on adrenocortical cells stimulating the production and release of cortisol^13^. Release of CRH is followed by enhanced secretion of ACTH from the anterior pituitary and cortisol from the adrenal cortex. Circulating cortisol feeds back at the PVN and anterior pituitary to inhibit the secretion of CRH and ACTH, respectively.

Since CRH is released into the hypothalamo-hypophyseal portal system, very little, if any, goes into the general circulation. But release of CRH is followed by enhanced release of ACTH. Indeed, when CRH is absent very little, if any, ACTH, is secreted^14^. It was on this basis that the present study measured the concentration of ACTH in the plasma of the rats to reflect release of CRH from the PVN. Whereas the concentration of ACTH in the plasma of control rats remained normal throughout the study period, that of experimental rats started rising soon after infection and then declined towards the end of the study period. This finding was in agreement with that of Mutayoba et al.^15^, who reported an initial increase, and then a rapid decline, in plasma ACTH concentration in *T. congolense*-infected rams than in the controls.

CRH and ACTH are important components of the hypothalamic-pituitary-adrenal (HPA) axis which, together with the arousal and autonomic nervous systems, constitutes the stress system. This system is activated during stress and produces the clinical phenomenology described as the stress syndrome.^16^ Secretion from the HPA axis exhibits two distinct activation patterns: circadian-dependent release, which is driven by the suprachiasmatic nucleus and is essential for maintaining normal energy balance; and stress-dependent release, which follows internal or external challenges^17^,^18^. During trypanosome infection, as in the present study, the stress-dependent release overrides the circadian-dependent release. This is because the trypanosome infection causes stress to the animal. This activates the stress system and accounts for the increased level of ACTH in the experimental rats for the first three weeks post-infection.

Continued presence of trypanosome, and/or factors released by the trypanosome, continuously stimulated the PVN which, in turn continued secreting more CRH, and hence, more ACTH in circulation in experimental rats. The persistent stimulation may have ultimately made the neurons of the PVN to become exhausted causing their nuclei to become smaller in size compared to those of control rats. This could have caused the decrease in plasma concentration of ACTH in the experimental rats towards the end of the study period.

Histological changes in the anterior pituitary gland of experimental rats were observed in the present study. The nuclei of the pituitary corticotrophs could have initially become hypertrophied in response to continued stimulation following infection. They then became exhausted and shrunk towards the end of the study. This finding was in agreement with those of other investigators^15^,^19^ who also reported histological alterations in the anterior pituitary gland of trypanosome-infected sheep and cattle. The pituitary corticotrophs are the ones responsible for synthesis of ACTH, and histological changes in them are likely to lead to dysfunction in ACTH secretion. This could further explain the decline in plasma concentration of ACTH recorded in the experimental rats towards the end of the present study.

## Conclusion

This study has demonstrated that *T.b. brucei* infection causes an initial increase, and then a decrease, in concentration of plasma ACTH in infected rats. The infection also causes histological changes in the PVN and pituitary corticotrophs of the rats. These histological changes cause the PVN and the pituitary corticotrophs to produce less CRH and ACTH, respectively.
